# Design and Rationale of the ERA-CVD Consortium PREMED-CAD—Precision Medicine in Coronary Artery Disease

**DOI:** 10.3390/biom10010125

**Published:** 2020-01-11

**Authors:** Apurva Shrivastava, Vincenzo Marzolla, Henri Weidmann, Massimiliano Caprio, David-Alexandre Tregouet, Tanja Zeller, Mahir Karakas

**Affiliations:** 1 Clinic for Cardiology, University Medical Center Eppendorf, 20246 Hamburg, Germany; a.shrivastava@uke.de (A.S.); h.weidmann@uke.de (H.W.); t.zeller@uke.de (T.Z.); 2 German Center for Cardiovascular Research (DZHK), Partner site Hamburg/Kiel/Lübeck, 20246 Hamburg, Germany; 3 Laboratory of Cardiovascular Endocrinology, IRCCS San Raffaele Pisana, 00163 Rome, Italy; vincenzo.marzolla@sanraffaele.it (V.M.); massimiliano.caprio@sanraffaele.it (M.C.); 4 Department of Human Sciences and Promotion of the Quality of Life, San Raffaele Roma Open University, 00163 Rome, Italy; 5 INSERM, U1219 Bordeaux Population Health research center, University of Bordeaux, 33076 Bordeaux, France; david-alexandre.tregouet@u-bordeaux.fr

**Keywords:** PREMED-CAD, precision medicine, biomarker, coronary artery disease, myocardial infarction, clinical trial, ischemia, at-risk

## Abstract

Cardiovascular diseases (CVDs) comprise 45% of all deaths in Europe and causes 3.9 million deaths annually. Coronary artery disease (CAD) which includes myocardial infarction (MI) represents the most common form of CVD. A relevant proportion of MI cases seems preventable since reports claim that up to two-thirds of these patients exhibit symptoms suggestive for MI within 12 months prior to the acute MI event. An early identification of these at-risk subjects is necessary to manage an early and efficient treatment during the ischemic phase. The aim of the PRecision MEDicine in Coronary Artery Disease (PREMED-CAD) consortium is to apply a system medicine approach towards studying and identifying an ischemia specific ‘biomarker signature’ that improves the identification of individuals ‘at-risk’ for acute MI. The consortium will take an interdisciplinary and translational approach integrating knowledge from CAD epidemiology, imaging, bioinformatics, statistics and molecular biology, as well as existing phenotypic, blood-based and clinical biomarker data of distinct CAD and subclinical MI phenotypes. This biomarker signature will be validated through atherosclerosis-prone mouse models and human cohorts. The validated signature will be translated in a real-world clinical setting using an ongoing clinical trial comprising patients with subclinical ischemia. The aim of the knowledge obtained from this project is to aid in early MI detection and reduce the mortality and morbidity rate in these at-risk MI individuals.

## 1. Introduction

Cardiovascular disease (CVD) causes more deaths among Europeans than any other disease condition [1]. According to Global Burden of Disease database, there was an incidence of 6 million of individuals suffering acute coronary syndrome (ACS) in Europe in 2015. CVD is among the leading causes of death in Europe and the World Health Organization (WHO) has stated that 80% of the deaths are due to premature heart diseases are preventable with intervention at the correct time. It is estimated that up to two-thirds of these individuals consulted at the emergency room (ER), a specialized chest pain unit (CPU), or the general practitioner within 12 months prior to the acute event with symptoms suggestive for myocardial ischemia. Thus, an early identification is required for these at-risk subjects <12 month prior to the disease occurrence, in order to significantly reduce morbidity, mortality, and the burden on health and socioeconomic status.

Existing clinical tools to identify the initial (subclinical) stages of CVD include electrocardiography (ECG), echocardiography, measurement of troponin, magnetic resonance imaging (MRI), and elective coronary angiography. These tools often fail to take into consideration the myriad of risk factors associated to CVD that would aid in providing a timely intervention. As evidenced by data from the American National Cardiovascular Data Registry, elective coronary angiography shows low diagnostic yield [2]. Out of a total of 398,978 patients undergoing coronary angiography, only 37.6% had an obstructive CAD [2]. Moreover, in this registry, non-invasive tests like ECG, ergometry, and stress echocardiography showed no incremental value. Despite the well-established knowledge on CVD risk factors, current existing tools fail to adequately identify subjects prior to MI event. Recently, in order to improve the risk estimation of CAD, much attention has been given to the potential of circulating, blood-based biomarkers. Multiple studies evaluated the predictive value of biomarkers reflecting hemodynamics, micronecrosis, inflammation, lipids, neuro-humoral activity, and renal function beyond the classical risk factor models. In particular, the strongest additional predictive value has been shown for natriuretic peptides (NPs), high sensitivity assayed troponin (hs-Tn), and C-reactive protein (CRP). NPs are synthesized in response to cardiac wall stress in association with volume overload [3], while, troponin levels are usually increased because of cardiomyocyte necrosis [4]. CRP is a sensitive but a non-specific marker of inflammation which positively correlates with CVD incidence [5] and mortality [6].

## 2. Unmet Need and Rationale of PREMED-CAD

Within PREMED-CAD, a biomarker signature for ischemia prior to MI event will be derived using bioinformatics tools and its validation in animal models and human disease cohorts. In the final step, the validated biomarker signature will be translated into the ongoing clinical trial GRAY-ZONE to evaluate the clinical applicability of the biomarker signature for the early identification of at-risk subjects for ischemic coronary events. The aim of the PREMED-CAD consortium is to find biomarkers that have the ability to identify patients that are at-risk (<12 months) of developing MI. The groundwork of PREMED-CAD relies on an already existing extensive set of biomarkers, epidemiological cohorts with deep phenotyping and imaging data, biomaterials, and distinguished animal models. The target population are at-risk MI patients that show inconclusive symptoms of CAD and within whom diagnostic and therapeutic measures fall short.

## 3. Design and Methodology

### 3.1. Design

The PREMED-CAD consortium comprises of three participating partner institutions in Europe, the University Medical Center, Hamburg (Hamburg, Germany), INSERM Bordeaux (Bordeaux, France) and IRCCS San Raffaele Pisana (Rome, Italy). The partner institute in Hamburg is one of the largest German University hospital treating +20,000 CVD patients per year. Therefore, within this consortium, access to clinical cohorts will be provided by the German partner. The Italian partner holds extensive experience with animal models of atherogenesis and atherosclerosis and aims to validate and consolidate findings of the human cohorts. The French partner provides computational infrastructure and statistical and bioinformatical expertise to identify a concise risk signature out of the vast data available and being generated. All partners hold deep understanding and high-level expertise on the fundamentals and the characteristics of CVD and underlying atherosclerosis. Each participating institution contributes specific experience, materials and methods that enable a systemic investigation of CVD mechanisms leading to a validated biomarker signature that improves early MI at-risk detection. The diversity of expertise ranges from computational to clinical-molecular and molecular-physiological knowledge. A large spectrum of molecular biology and human resources along with the ability to translate the final biomarker signature through a clinical trial will result in the maximal use of all available data.

**Figure 1:**
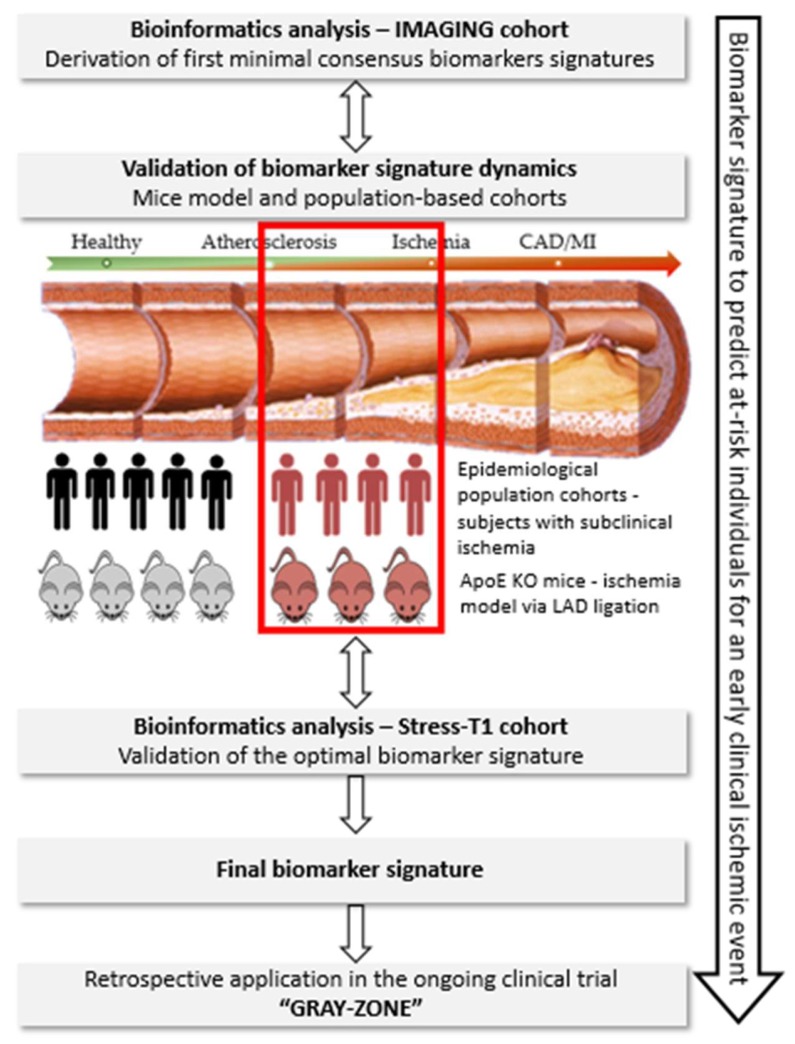
Schematic presentation of the methodology that will be followed to achieve the optimal biomarker signature in the PREMED-CAD consortium. Target disease population: the subclinical disease stage, immediately (<12 months) before the occurrence of coronary events will be studied. Work-flow of experiments: through bioinformatics tools and validation in murine models and epidemiological cohorts, the biomarker signature for subclinical ischemia in CAD will be identified and validated in the ongoing clinical trial GRAY-ZONE.

Within PREMED-CAD, a biomarker signature specific to ischemia will be derived (Figure 1). For this purpose, deep-phenotyping data, blood biobank and cardiac stress-MRI test are available for at-risk MI patients from the IMAGING cohort, *n* = 200 (signature derivation cohort) and Stress-T1 cohort, *n* = 200 (signature validation cohort). The derived ischemia specific biomarker signature will be compared to patients without ischemia within the IMAGING and Stress-T1 cohort. Additional validation will be performed in ischemia animal models and optionally in the large population-based study—the Hamburg City Health Study (400 ischemia-MRIs). Ultimately, the validated biomarker signature will be translated in an ongoing clinical trial GRAY-ZONE to evaluate the clinical applicability of the biomarker signature for the early identification of at-risk subjects for ischemic coronary events.

### 3.2. Methodology

#### 3.2.1. Derivation of the Optimal Biomarker Signature

Derivation of first minimal consensus biomarker signature will be performed in the IMAGING cohort data by capitalising on already identified candidate biomarkers (including miRNAs, troponin, natriuretic peptides, C-reactive protein (CRP) and metabolites). The disease signature will be derived when the data from the diseased cohort is compared to healthy individuals. For the analysis, we will apply generalised additive models [7] coupled with backward selection procedures to identify the most relevant linear and non-linear predictors of CVD. Discriminative performances and adjustment for optimism will be performed on each cohort. Subsequently, different correlation networks [8], unsupervised clustering and machine/deep learning methodologies [9,10,11] will be applied to identify new biological partners to these candidates. These data will also be integrated into knowledge-based biological databases such as KEGG, Reactome, Gene Ontology database and other publicly available “omics” resources. In the final step, identified markers will be combined to determine an optimal biomarker signature between performance of the prediction model and clinical usefulness. Regression coefficients from the multivariate regression models will be transformed into the components of a risk score. Sensitivity, specificity, positive predictive value (PPV) and negative predictive value (NPV) for several cut-off scores will be calculated.

#### 3.2.2. Validation of Biomarker Signature

To validate the biomarker signatures derived from bioinformatics analyses, independent measurements will be performed in experimentally characterized murine models as well as in independent human epidemiological cohort. This will help to better understand the dynamics of our identified biomarker signature since we evaluate it in disease models mirroring the transformation from healthy stage to disease stage.

**Figure 2:**
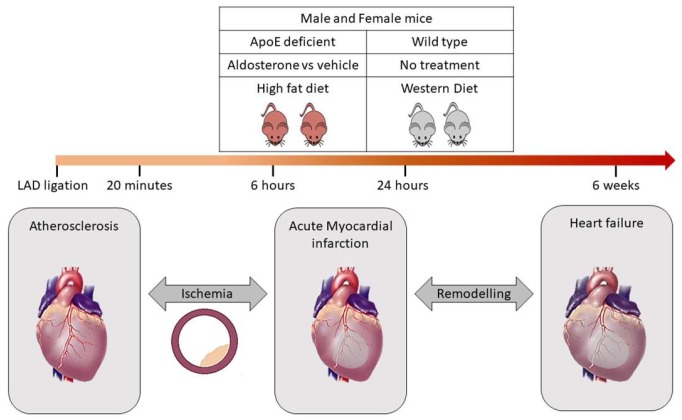
Validation of bioinformatics derived biomarker signature through mice model. The ApoE knockout mice subjected to high fat diet under the influence of aldosterone or vehicle will be compared to wildtype mice subjected to western diet. LAD ligation is performed for both ApoE-/- and wildtype mice for different time periods such as 20 min, 6 h, 24 h and 6 weeks. This will help to distinguish the signature specific for ischemia prior to MI event.

Characterisation in experimental murine models will be performed in apolipoprotein E-deficient knockout (ApoE-/-) mice. A remarkable body of evidence has shown that ApoE-/- mice represent a suitable animal model to study mechanisms underlying the atherosclerosis process. In addition, treatment of such mice with aldosterone allows to study vascular alterations occurring in atherosclerosis [12,13]. The influence of aldosterone + proatherogenic high fat diet (HFD) will be compared to aldosterone + western diet in both ApoE-/- mice and wild-type mice (i.e., four groups, each *n* = 10 female and male). In these four groups, the spectrum of atherosclerosis progression (from ischemia to MI) will be achieved by occlusion of the left anterior descending (LAD) since LAD ligation represents a valuable experimental model of MI and ischemia (Figure 2).

Therefore, the mice will be subjected to different conditions that accurately represent the dynamics of progression of CAD prior to MI. LAD occlusion will be performed for different time periods such as 20 min, 6 h, 24 h and 6 weeks. Such a model is suitable to obtain a broader overview of the difference in signature prior to the MI event and validate very early markers of acute MI before the occurrence of tissue necrosis [14]. To address the gender issue, both female and male mice (*n* = 15 in each group) with ApoE-/- will be chosen, since ApoE-/- male mice develop an atherosclerotic plaque within 4–8 weeks of aldosterone treatment and concomitant feeding with proatherogenic HFD, whereas the female mice develop increased plaque development even in the absence of aldosterone treatment. Samples are collected to allow for measurement of biomarkers. All animal experiments are approved by the Italian National Institutes of Health Care and Use Committees, Rome, Italy (493/2016-PR, 05/17/2016, Prof. Massimiliano Caprio, MD, Ph.D)

In parallel to the validation of the biomarker signatures in murine models, additional epidemiological cohorts (Stress-T1, population-based cohort) will serve for independent validation and biomarkers will be measured. For this, serum/plasma samples from patients with early ischemia i.e., Stress-T1 cohort bio-bank will be used. Similar to the IMAGING cohort, Stress-T1 is a cardiac stress-MRI cohort that was specifically designed in order to find new blood-based biomarkers predicting myocardial ischemia. Both cohorts share the same inclusion criteria (age above 18 years, patients capable of giving informed consent, clinical indication for routine cardiac stress-MRI), and exclusion-criteria (acute myocardial infarction, known untreated coronary heart disease, known myocardial ischemia as assessed by stress-echo, scintigraphy, or similar procedures).

## 4. Translational Aspect—GRAY-ZONE

The current literature shows that in patients presenting with chest pain, high-sensitive troponin levels above the 99th percentile are indicative for future cardiovascular events, even when acute coronary syndrome was ruled out. Most of these non-ACS-patients are discharged without specific/ preventive therapy (anti-platelet or anti-lipid), although ‘positive’ troponin values (any value at any time during hospitalization above the 99th percentile) seem to clearly indicate underlying myocardial ischemia. In summary, there is an unmet need with a huge potential to reduce mortality and morbidity in ER patients by specific therapy. GRAY-ZONE is an ongoing clinical trial, fully financed by the German Research Foundation, and initiated by the German partner. A total of 3000 troponin positive patients presenting at ER/ CPU with acute chest pain, but ruled-out ACS, will be assigned randomly to Aspirin and/ or Atorvastatin versus placebo (2 × 2 factorial design). The primary endpoint is a composite of non-fatal stroke, non-fatal MI and cardiovascular death. The final biomarker signature, which will be derived within the PREMED-CAD project will be translated and validated retrospectively in GRAY-ZONE. Therefore, we will be able to assess whether this signature is capable of predicting those subjects developing a clinical ischemic event during the 12-months follow-up. Bio-banking in the GRAY-ZONE trial is available at baseline and at 12-months follow-up. This will allow us to investigate the dynamics within the identified biomarkers and final biomarker signature; and also in the clinical event rate in PREMED-CAD-biomarker positive versus PREMED-CAD-biomarker negative patients of the three active, well-tolerated therapies (Aspirin, Atorvastatin, Aspirin combined with Atorvastatin). This project directly paves the way to personalized and biomarker-guided therapy.

## 5. Challenges and Risks

Because of the complexity of the project including the translational approach, this project has explicit challenges and risks. The project is based on the ‘stratification tool’—the biomarker signature—which allows the identification of subjects at early risk for myocardial infarction. The translational quality of the biomarker signature will be evaluated by the application in the randomized GRAY-ZONE clinical trial. This will allow, by immediate post-hoc analyses, to determine the diagnostic value of the biomarker signature (‘at risk’ = MI within 12 months). Moreover, the exclusive access to this clinical trial allows the therapeutic evaluation of the biomarker signature, since the ischemic subjects of the clinical trial will be assigned to four different medication therapies. This implies that the early stage of biomarker derivation is crucial for all following steps. There is no immediate clinical endpoint in our trial—a weak and insufficient signature will endanger all following steps and the translation into the clinical trial. On the other hand, this collaboration bears the potential to carry a biomarker signature from early development up to ultimate diagnostic application in an ongoing clinical trial, and even towards personalised medicine application using therapy-response analyses. Therefore, this high potential project bears the abovementioned risk and justifies the endeavours and resources.

## 6. Expected Outcome

Given that CVD represents the most important cause of morbidity and mortality, results of the PREMED-CAD consortium have the potential to reduce morbidity and mortality by an early detection and timely therapy of at-risk subjects. Consequently, the results obtained herein will improve and append the knowledge on possible precision medicine approaches for MI patients.
